# A Nursing Centre for the Empire

**Published:** 1920-10-23

**Authors:** 


### THE MATRONS' AND SISTERS' DEPARTMENT.

### A NURSING CENTRE FOR THE EMPIRE.

The news we are privileged to announce on p. vi will send a thrill of pride and pleasure through nursing circles in every part of the kingdom and far beyond. The College of Nursing now for over four years has gone quietly and steadily about its business, rallying British nurses for the formation of a great confederation. Nearly twenty thousand have obeyed the call and have fallen into line with wonderful unanimity, showing abundant evidence of original and spontaneous ability in the organisation of their profession. The time had come for some outward and visible sign that the nursing profession had achieved unity, and with the time has come also the woman with insight and magnificent generosity to crown the enterprise so finely conceived.

Viscountess Cowdray has long been inspired with admiration for nursing ideals and intense belief in them. By the noble gift which she and Lord Cowdray have lately bestowed on the nursing profession they have added yet another token of far-sighted sympathy with nurses and supplied the source of strength the College conspicuously lacked, a force they could not otherwise have attained for many years. The pressing need of the nursing service has long been a recognised centre to which nurses could turn from all parts of the world, and which the public could recognise as the headquarters of the profession.

The fine mansion in Cavendish Square which has now passed into the possession of the College of Nursing lies in the midst of the district specially appropriated to medical and nursing activities. The square is one of the finest in London, and in the seclusion afforded under the trees it is hardly possible to realise that the roar of Oxford Street resounds a few yards away. The uses to which these noble premises will be put are twofold, and both are essential to the full development of the College as a world-wide institution. In the first place, they will constitute a Club for the purposes of the London Centre of the College. It has long been evident that the London centre stood in a peculiar relation to all the other centres, that it had duties and responsibilities apart from the interests of its own members, and that it must be charged with the special honour and privilege of representing the College in the eyes not only of the other centres, but of guests from overseas who visit our shores. In the somewhat cramped quarters which the London centre now occupies it has done its part well. Removed to Cavendish Square it will be in a position to exercise its functions with a dignity not hitherto possible.

We can forecast many delightful festivities and reunions among nurses at home and from different climes, made possible by the magnificent dining and reception rooms, which are unsurpassed by any Ladies' Club in London. And we find it hard to believe that any nurse will ever for the future be able to complain of loneliness in the great Metropolis. No. 20 Cavendish Square will, we anticipate, be the rallying point for the nurses of the Empire collectively and individually, and hospitality its keynote. We should rejoice to hear that one or two bedrooms could be reserved always for members from overseas arriving in England.

But there is a second purpose in Lady Cowdray's gift. The College needs very spacious accommodation for its increasing staff, for its valuable records, for the instruction of entrants to the profession, for committee meetings and councils. We look to see a building on the vacant site behind planned to meet all requirements and worthy to be called the centre of the nursing profession.

The Central Hall of the College should be beautiful, to match the ideals of the great foundress of the profession. Nursing stands, so it would seem, on the brink of developments far in advance of anything yet achieved. Here should be a centre of professional study and progress. Here should be a centre for discussion and adapted for great gatherings. Here smaller conference rooms should be found dedicated to the great names in nursing story. Here in statue and portrait should be many notable figures whose memory is perpetuated by the profession they helped to build.

